# The Vacuolar Protein 8 (Vac8) Homolog in *Cryptococcus neoformans* Impacts Stress Responses and Virulence Traits Through Conserved and Unique Roles

**DOI:** 10.3390/jof11120877

**Published:** 2025-12-11

**Authors:** Peter V. Stuckey, Julia Marine, Meghan Figueras, Aliyah Collins, Felipe H. Santiago-Tirado

**Affiliations:** 1 Department of Biological Sciences, University of Notre Dame, Notre Dame, IN 46556, USA; pstuckey@nd.edu (P.V.S.); acolli92@vols.utk.edu (A.C.); 2 Eck Institute for Global Health, University of Notre Dame, Notre Dame, IN 46556, USA; 3 Warren Center for Drug Discovery, University of Notre Dame, Notre Dame, IN 46556, USA

**Keywords:** *Cryptococcus neoformans*, fungal vacuole, fungal pathogen, armadillo repeats

## Abstract

Functionally similar to a plant vacuole or a mammalian lysosome, the fungal vacuole plays a vital role in many cellular processes. Most studies of the vacuole have been performed in the nonpathogenic yeast *Saccharomyces cerevisiae*; however, the vacuole in pathogenic fungi has recently been implicated in host invasion in both plants and mammals, highlighting an important role for the vacuole in pathogenesis. Here, we report that deletion of *C. neoformans* vacuolar protein 8 (*VAC8*) results in fragmented vacuole morphology, impairment of vacuolar fusion, and inability to form titan cells. Additionally, absence of Vac8 results in defective growth at high temperature and in the presence of caffeine, suggesting a defect in cell wall signaling. Interestingly, despite aberrant vacuole morphology, *vac8*Δ is slightly more resistant to fluconazole treatment, and displays increased resistance to hydrogen peroxide, suggesting the irregular vacuole morphology does not impair vacuolar function. Like *S. cerevisiae* Vac8, *C. neoformans* Vac8 is comprised of armadillo repeat regions which form alpha helices that fold to form a superhelix, allowing for increased protein–protein interaction. Many of the known binding partners of *S. cerevisiae* Vac8 are not present in the *C. neoformans* genome, suggesting novel functions for Vac8 in this fungus. Notably, deletion of *VAC8* affected some virulence traits, providing support for targeting the fungal vacuole as a potential therapeutic intervention.

## 1. Introduction

The incidence of serious and/or invasive fungal infections has been increasing in the last few decades, with overall mortality rates >20% [1]. Despite this, there have been few advances in available antifungal treatment options, with most targeting cell wall components or ergosterol, a cholesterol-like lipid found only in fungi [2]. Additionally, these medications are complicated by off-target toxicity, restrictive formulations, limited activity, issues with availability and supply, and the emergence of antifungal resistance [3,4]. Therefore, morbidity and mortality rates from systemic fungal infections remain incredibly high despite a decrease in the human immunodeficiency/acquired immunodeficiency syndrome (HIV/AIDS) population, which are the most susceptible to these infections [5]. This highlights the need for novel antifungal therapies and the identification of potential new drug targets.

The fungal vacuole is a dynamic organelle that reacts to a variety of environmental stresses and changes its morphology accordingly. Functionally similar to a plant vacuole or a mammalian lysosome, the fungal vacuole plays a vital role in the degradation of intra- and extracellular substrates, recycling of nutrients, metabolite storage, and detoxification [6,7]. To perform this diverse set of functions, the vacuolar membrane is constantly undergoing coordinated fusion and fission events to respond to changes in the environment. Most studies of fungal vacuoles have been performed in the nonpathogenic yeast *Saccharomyces cerevisiae*, where multiple genes involved in vacuolar functions have been characterized (known as *VAC* genes). In *S. cerevisiae*, *VAC8* (Sc*VAC8*) has been extensively studied, given that it encodes the only Armadillo (ARM) repeat-containing protein in yeast [8]. The protein structure of ARM repeats mediate protein–protein interactions with a variety of conformations, allowing *VAC8* to be involved in many cellular roles, including vacuole fusion, vacuole inheritance, and autophagosome formation, among others [8,9]. Therefore, much of what we assume to know about the vacuole in pathogenic fungi comes from *S. cerevisiae*, despite there being large genetic differences. We do know that at least some of these vacuolar functions are critical for some pathogenic species to survive, invade mammalian hosts, and cause disease [10,11,12].

In human fungal pathogens, the role of the vacuole in basic cell biology and during infection is largely unexplored, but recently has been shown to play a critical role in supporting host invasion in mammals. *Candida albicans* and *C. neoformans* mutants with greatly impaired vacuole function do not cause lethal disease in mouse models of disseminated infection [10,11,12]. In *C. neoformans*, deletion of a vacuolar proton pump, *VPH1*, leads to defects in major virulence factors, including capsule production, reduction in thermotolerance, laccase function, and urease secretion [13]. This leads to the complete loss of virulence in a mouse model of disease. Likewise, in other fungal pathogens, like *Candida glabrata* and *Histoplasma capsulatum*, normal vacuolar function has been implicated in the ability to cause disease in mouse models [14,15].

Particularly for *C. neoformans*, the interactions with host immune cells are critical for determining the outcome of the infection, and can play a key role in the dissemination to the central nervous system [16,17]. For example, clinical, in vivo, and in vitro data have demonstrated a strong correlation between phagocytosis of *C. neoformans* by macrophages and a negative outcome for patients [18,19,20]. Isolates with high rates of in vitro phagocytosis and high intracellular proliferation correlated with patient death (despite treatment, they all died at 3 months) [19]. Similarly, increased rates of in vitro phagocytosis correlated with high fungal burden on the brain, which increases the risk of mortality [18,19,20]. Along these lines, our lab previously sought to screen a single gene deletion collection of *C. neoformans* for mutants that display altered phagocytosis in vitro by THP-1 human macrophages [21]. One of the identified mutants, missing the gene *PFA4* that encodes an S-acyl transferase that catalyzes lipid modifications of proteins, displayed severe defects in a wide variety of cell stressors and was completely avirulent in an intranasal mouse infection model [13,21]. In an effort to mechanistically explain the phenotypes, we used click chemistry to identify the specific proteins modified by Pfa4, in the process determining the first “palmitoylome” of any fungal pathogen [21]. One of the substrates was the protein encoded by CNAG_00354, the homolog of Sc*VAC8*, which is known to be palmitoylated in both *S. cerevisiae* and *Toxoplasma* species [22,23]. Given the pleiotropic effects exhibited by the *pfa4*Δ mutant and the known involvement of Sc*VAC8* in vacuolar functions, we wondered if *VAC8* would have similar functions in *C. neoformans*. If true, vacuolar dysfunction might be one of the factors contributing to the lack of virulence in *pfa4*Δ.

Here, we report that *pfa4*Δ shows vacuole defects similar to those shown by the deletion of *VAC8* (*vac8*Δ). ScVac8 is palmitoylated and myristoylated, which is required for tethering to the vacuole membrane in *S. cerevisiae* [24]. Given that *C. neoformans* Vac8 also contains the same lipid-modified residues as ScVac8, it would be expected that, in the *pfa4*Δ mutant, Vac8 would be mislocalized, thus affecting vacuolar function. To carefully examine the effects of *vac8*Δ, we visualized vacuole morphology in wildtype and *vac8*Δ *C. neoformans* strains, finding that the deletion of *VAC8* leads to an aberrant and fragmented vacuolar structure. Additionally, the *vac8*Δ strain displays increased sensitivity to caffeine, suggesting a defect in cell stress signaling, but also increased resistance to high levels of oxidative stress (H_2_O_2_) compared to WT strains. Notably, *vac8*Δ strains show increased resistance to fluconazole (FLC) but no change to amphotericin B (AmB). The pleiotropic phenotypes of *vac8*Δ are not surprising given the multiple binding partners that Vac8 might have. The multiple ARM repeats allow Vac8 to mediate multiple processes, some of which are important for virulence. Interestingly, despite histological and gross tissue differences in a murine cryptococcosis model of infection, there were no differences in survival between wildtype-infected and *vac8*Δ-infected mice. Nevertheless, our work supports the notion that disrupting vacuolar functions might be an effective, novel therapeutic intervention.

## 2. Materials and Methods

### 2.1. Strains, Cell Lines, and Growth Conditions

All fungal strains used are in the KN99 background [25]. Cells were thawed from −80 °C stocks every 6 months and struck onto YPD plates, allowing for 48 h of outgrowth at 30 °C prior to storage at 4 °C for up to a month before being re-plated. Cells were grown in YPD liquid media overnight at 30 °C with shaking (225 rpm). Cultures were then diluted back to OD_600_ of 0.2 and allowed to double until OD_600_ reached 0.5–1.0, unless otherwise stated.

### 2.2. In Silico Sequence Analysis

Sequences of proteins analyzed in this paper were obtained from NCBI (www.ncbi.nlm.nih.gov) or FungiDB (fungidb.org). To search for Vac8 homologs, the Sc*VAC8* and Cn*VAC8* sequences were analyzed with NCBI BLAST v2.16.0 against other common fungal pathogens. Selected proteins representing Basidiomycota, Ascomycota, and Mucorales were utilized to create a phylogenetic tree. Phylogenetic analysis was performed using the maximum likelihood method in MEGA11 software, and confidence intervals were determined from 1000 bootstraps, with bootstrap scores shown at all nodes [26]. The tree shown is the bootstrap consensus tree.

Protein domain analysis was performed using Interpro and Pfam databases. Protein structures were predicted and visualized using Alphafold2 for *C. neoformans*, *S. cerevisiae*, and *C. albicans*.

Palmitoylation was determined using the palmitoylation prediction software GPS Palm [27], and myristylation was determined using software [28]. All relevant information regarding the protein sequences analyzed, predictive modification values, and protein ID numbers are provided in Supplemental Table S1.

### 2.3. Drug Susceptibility Assays

The MICs of amphotericin B and fluconazole were determined on RPMI plates with Epsilometer test strips (E-test strips; Liofilchem Thermo Fisher, Waltham, MA, USA), as described in [29]. 5  ×  10^4^ log-phase cells in 200 μL PBS were plated on RPMI agar plates, spread evenly across the plate, and allowed to dry before application of an E-test strip. Plates were incubated at 37 °C and 5% CO_2_, and imaged after 72 h.

### 2.4. Stress Plate Phenotyping

Strains were grown overnight in YPD at 30 °C with shaking (225 rpm) to an OD600 of 0.5–1.0. Cells were washed with PBS and the concentration was adjusted to 10^7^ cells/mL. Cultures were then serially diluted 10-fold in PBS and spotted (5 μL) onto solid YPD ager medium, incubated at 30 °C, unless otherwise noted, for 3 days, and imaged daily. The response of various *vac8*Δ mutants was tested under the following conditions: 1 M sodium chloride (NaCl); 0.75 mg/mL caffeine; 0.3% sodium dodecyl sulfate (SDS); 0.5 mg/mL calcofluor white (CFW); 2 mM and 4 mM hydrogen peroxide (H_2_O_2_). YPD and RPMI plates were also tested at 37 °C.

### 2.5. Fungal Genomic Manipulation

The commercially available CNAG_00354 single gene deletion mutant was obtained from the *Cryptococcus* deletion collection (Fungal Genetics Stock Center; www.fgsc.net/ (last accessed 3 December 2025)). To complement this strain, the coding sequence of *VAC8* was amplified via PCR with primers containing restriction digest sites for FseI and NheI. The PCR product was then digested with FseI and NheI in rCutsmart buffer and ligated into plasmid pDC-mRuby3-neo, which was derived from pGWSK7 (mRuby3) [30]. This plasmid contains a histone 3 promoter and mRuby3 tag at the C-terminus. The entire cassette, including neomycin resistance, was amplified via PCR using primers with 50 bp homology to the safe-haven 2 (SH2) region [31]. Cas9 was amplified from pBMH2403_CnoCas9, and the guide was amplified from a derivative of pBHM2329 [32]. DNA was then electroporated into the *vac8*Δ mutant using standard electroporation settings. Briefly, cells were grown overnight in YPD, then diluted back to OD600: 0.2 and allowed to grow until the mid-log phase. Cells were then washed twice with ice-cold diH_2_O and resuspended in 10 mL of electroporation buffer (EB) with DTT. Cells were then pelleted and resuspended in 250 μL EB without DTT; following this, they were electroporated in a 2 mm gap cuvette with 45 μL of cells, 500 ng of Cas9, 350 ng of sgRNA, and 2000 ng of repair construct at 2000 V, 25 μF, and 200 Ω. Cells were immediately supplemented with YPD and incubated at 30 °C, with rotation for 2 h prior to plating on YPD + Neo (200 μg/mL) plates, and incubated at 30 °C for 72 h. Complementation was confirmed by amplifying the entire SH2 region and sequencing.

### 2.6. Titan Cell Induction and Quantification

Cells were grown overnight in 5 mL of YNB at 30 °C with shaking (225 rpm). Log-phase cells were harvested and washed 2X with PBS and counted using a Bio-Rad TC10 cell counter. Cell density was then adjusted to 1000 cells/mL in 10 mL of 10% heat-inactivated fetal bovine serum (FBS) (Corning Life Sciences, Corning, NY, USA) in PBS in a T25. Flasks were placed in a 37 °C environment with 5% CO_2_ and incubated for 48 h before being washed and imaged. At least 10 fields of view were randomly imaged to avoid biases. Cell size, capsule size, and total cell size were quantified manually in FIJI using the average of four measurements per cell.

### 2.7. Vacuole Induction and FM4-64 Staining

To visualize the vacuole of *C. neoformans* strains, cells were stained with FM4-64X (Thermo Fisher Scientific, Waltham, MA, USA) and 100 ng/mL CFW (Sigma Aldrich, St. Louis, MO, USA) to highlight the cell wall. Briefly, cells were grown under specified conditions, washed 2X with PBS, and resuspended in 100 μL of PBS. An amount of 2 µL of FM4-64 (with a final concentration of 32 µM) was added and incubated at 30 °C for 30 min with rotation. Cells were then washed 2X with PBS and resuspended in 50 μL of PBS. The cells were then imaged under a Zeiss Axio Observer 7, with an Axiocam 506 mono camera and a FM4-64 long-pass filter set, using 100X/1.4 plan-apo oil objective. At least 10 fields of view were randomly imaged to avoid biases. To induce the large vacuole and test for vacuole fusion, cells were grown overnight in YPD; then, they were washed and grown in YPD + 1 M sorbitol at 37 °C for 4 h prior to staining and imaging, as performed previously [33].

### 2.8. Macrophage Uptake Assay

Phagocytic index assays were performed as previously described [21], with small modifications. Briefly, THP-1 human monocytes were differentiated into macrophages using 0.16 mM phorbol 12-myristate 13-acetate (PMA) for 48 h before recovering in complete media for 24 h. Macrophages were infected at a multiplicity of infection (MOI) of 1 with the indicated yeast cells, which were opsonized in pooled human serum (40% serum and 60% PBS) for 30 min at 37 °C. Then, they were stained with 250 μg/mL Lucifer Yellow (Sigma Aldrich, St. Louis, MO, USA) for 20 min at room temperature with shaking. This co-culture was incubated for 1 h at 37 °C with 5% CO_2_ before being washed a processed, as performed previously [21]. Each 96-well plate was imaged in a non-biased manner and analyzed using Cell Profiler to remove potential bias. The phagocytic index was calculated as the number of internal fungi per 100 THP-1 cells.

### 2.9. Mouse Virulence Study

Female A/J mice, 5–6 weeks old (The Jackson Laboratory, Bar Harbor, ME, USA), were used in this study. All animals had free access to food and water. All testing and treatment of the mice in this study was performed in accordance with the University of Notre Dame and its Animal Care and Use Committee under the protocol number 23-05-7918. Briefly, fungal strains were cultured overnight in YPD to mid-log phase, collected, and washed and diluted to 1.25 × 10^6^ cells/mL in DBPS. Mice (between 12 and 19) were intranasally infected with 40 μL of the cell solution (5 × 10^4^ cells) or 40 μL of DPBS under anesthesia. Animals were monitored daily and sacrificed at set timepoints of 3, 7, and 14 days post-infection (between 2 and 4 mice), or when they lost >20% of their peak weight (between 4 and 9 mice). To quantify organ fungal burden, the lungs, brain, and spleen were homogenized using a VWR 200 homogenizer and plated on YPD + Ampicillin agar. After 48 h, CFUs were enumerated.

### 2.10. Histology

Female A/J mice (The Jackson Laboratory, Bar Harbor, ME) were infected via the intranasal route, as described above, with 5 × 10^4^ CFUs of either WT (KN99α), the *vac8*Δ mutant strain, the complemented *vac8*Δ:*VAC8* strain, or with sterile DPBS (mock). Mice were sacrificed at predetermined timepoints (3, 7, or 14 days post-inoculation, and at TOD) by isoflurane overdose, followed by cervical dislocation. Lungs were perfused with 10% neutralized formalin and subsequently paraffin-embedded, sectioned, mounted, and stained with H&E at the University of Notre Dame Histology Core facility. H&E-stained slides were imaged at 10× and 40× using an inverted Zeiss Axio Observer 7.

### 2.11. Statistical Analysis

All data were tested for statistical significance using Prism 10.5.0 (GraphPad Software). Information regarding specific statistical tests, number of replicates, number of cells/events quantified, and degree of significance are provided in the captions of each figure. The threshold of significance was set at *p* < 0.05.

## 3. Results

### 3.1. Deletion of Both PFA4 and CNAG_00354 Causes Vacuolar Fragmentation

**Figure 1:**
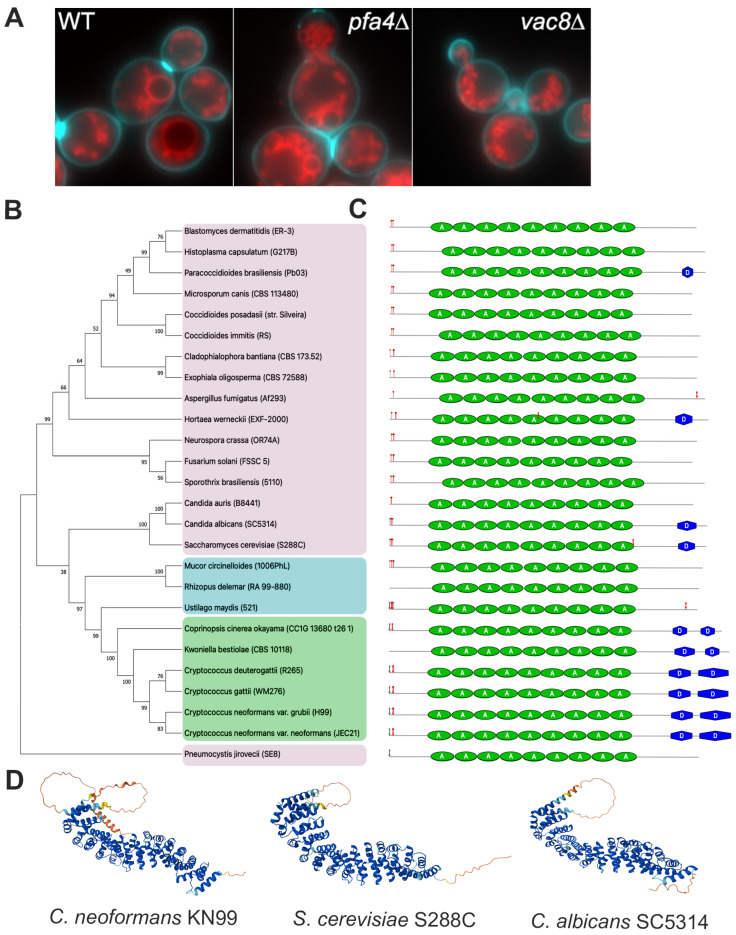
CNAG_00354 is a true homolog of Sc*VAC8*. (**A**) Vacuole localization and morphology in wildtype, strains lacking palmitoylation of Vac8 (*pfa4*Δ), or lacking Vac8 itself (*vac8*Δ). Vacuoles were stained with FM4-64 [37] and the cell outline with calcofluor white (blue). (**B**) Phylogenetic tree depicting the Vac8 amino acid sequence alignments in many clinically relevant fungi. Node support is represented as a percentage of 1000 bootstrapping iterations. Magenta shading: ascomycetes; blue shading: Mucorales; green shading: basidiomycetes. (**C**) Graphic representation of protein structure based on InterPro analysis of amino acid sequence shown to scale, relative to other Vac8 proteins. 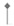 indicates predicted myristylation site, 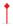 indicates predicted palmitoylation site, green ovals represent armadillo repeat regions, and blue hexagons represent predicted disorder regions. (**D**) AlphaFold predictions of *C. neoformans* (AlphaFold ID: AF-J9VIT9-F1), *S. cerevisiae* (AlphaFold ID: AF-P39968-F1), and *C. albicans* (AlphaFold ID: AF-Q59MN0-F1) Vac8.

Since CNAG_00354 shows homology to Sc*VAC8*, a key vacuole regulatory protein involved in vacuolar inheritance and fusion, nuclear–vacuole junctions, and autophagy, among others [34], we wondered if CNAG_00354 was also involved in vacuolar function. We used the lipophilic dye FM4-64 to visualize the vacuole of WT, *pfa4*Δ, and *cnag_00354*Δ grown under standard media conditions (YPD at 30 °C). We saw distinct but overlapping phenotypes (Figure 1A). In WT cells, there are usually 1–2 large vacuoles, with few tubules or smaller vesicles visible. In the *cnag_00354*Δ mutant, the vacuole was completely fragmented into small vesicles without tubules, while in *pfa4*Δ, there was an intermediate phenotype. Notably, there was no obvious vacuole inheritance defect, as we could see vacuoles present in bud and daughter cells, suggesting that CNAG_00354 is either not performing that function, as in *S. cerevisiae*, or there are redundant pathways in *C. neoformans* to ensure vacuole inheritance.

### 3.2. CNAG_00354 Is a True Ortholog of Saccharomyces cerevisiae VAC8

Given the similar but not identical phenotypes relative to *S. cerevisiae*, we sought to determine if CNAG_00354 is a true ortholog of Sc*VAC8* by performing reciprocal BLAST-P searches. We found a 1:1 relationship when using CNAG_00354 to search the *S. cerevisiae* genome or Sc*VAC8* to search the *C. neoformans* genome, confirming that CNAG_00354 (from now on named *VAC8*, following nomenclature guidelines [35]) is a true ortholog.

Next, we performed a phylogenetic analysis to determine sequence similarity and how widespread this protein is in the fungal kingdom (Figure 1B). The basidiomycetes, ascomycetes, and Mucorales species all cluster together within their respective groups, while the basidiomycetes and Mucorales appear to be more closely related than ascomycetes are to either clade. This clustering indicates that *VAC8* is well conserved in the fungal kingdom, but there are substantial differences from Ascomycota, including *S. cerevisiae*, which most of our fungal knowledge is based on. This suggests there may be both similar as well as unique functions of *VAC8* in *C. neoformans* and other basidiomycetes or Mucorales compared to ascomycetes.

In *S. cerevisiae*, Vac8 has numerous lipid modifications, including an N-terminal glycine that is myristoylated and three cysteines near the N-terminus that are palmitoylated, allowing the protein to anchor the fungal vacuole [34]. AlphaFold prediction indicates a highly conserved structure between ScVac8, CnVac8, and *Candida albicans* Vac8, including the 12 ARM repeats with an intrinsically disordered region near the N-terminus, with glycine and cysteine residues and a large disordered loop at the C-terminus (Figure 1D). Using the palmitoylation prediction software GPS Palm [27] and myristylation software [28], we investigated the likelihood of the cysteines in CnVac8 being palmitoylated and the N-terminal glycine being myristoylated. In ScVac8, the cysteine residues at positions 4, 5, and 7 are known to be palmitoylated (Supplemental Table S1). CnVac8 has cysteine residues in positions 8 and 9 that are also predicted to be palmitoylated (Supplemental Table S1). In congruence with the phylogenetic tree, *Cryptococcus gattii* has cysteine residues in positions 8 and 9, with predicted palmitoylation values of 0.9714 and 0.9667. *Ustilago maydis* has cysteine residues in positions 5, 7, and 8, with predicted palmitoylation values of 0.9677, 0.9396, and 0.9300, respectively, while the glycine is also myristoylated, with a score of 0.9572 (Figure 1B,C). Taken together, these data indicate that the overall protein domains and modifications of *VAC8* are conserved across the fungal kingdom, and that CnVac8 is a true ortholog of ScVac8.

Vac8 is an ARM repeat protein, comprised of a characteristic repetitive amino acid sequence of approximately 42 residues. These residues are often tandemly repeated, where each repeat gives rise to a pair of alpha helices which form a hairpin structure. Crystal structures have revealed ScVac8 has 11 complete ARM repeats and an incomplete 12th ARM repeat, which form alpha helices that fold to form a superhelix, allowing Vac8 to interact with a variety of binding partners [36]. This diversity in Vac8–protein interactions is what allows it to carry out diverse cellular functions. To this end, we explored the *C. neoformans* genome for orthologs of known ScVac8 binding partners. Many of the main known binding partners of ScVac8, including Nvj1, Vac17, Atg13, and TCO89, do not have homologs in *C. neoformans*, while Tao3 (transcriptional activator of *OCH3*), involved in apical bud growth and morphogenesis, is one of the few proteins with a potential homolog (Table 1).

**Table 1:** Analysis of Vac8 binding partners in *C. neoformans*.

Protein Name	Cellular Function	Present in *C. neoformans*	E-Value	Ref.
Nvj1p (YHR195W)	Piecemeal microautophagy of the nucleus	No		[38,39]
Vac17p (YCL063W)	Binding to Vac8 and Myo2 complex for vacuole inheritance	No		[38]
Atg13p (YPR185W)	Required for cytoplasm-to-vacuole (Cvt) pathway and autophagy	No *		[38]
Tco89p (YPL180W)	TORC complex subunit	No		[40]
Vid21p (YDR359C)	NuA4 histone acetyltransferase complex subunit	Yes CNAG_01591	7 × 10^−10^	[40]
Vab2p (YEL005C)	Vac8p binding	No		[40,41]
Tao3p (YIL129C)	Involved in cell morphogenesis and proliferation	Yes CNAG_03622	1 × 10^−79^	[40]

* Atg13 has a potential homolog in *C. neoformans* strain H99 (CNAG_00778), with an E value of 5 × 10^−4^. A BLASTp E-value cutoff of 1 × 10^−5^ was used as a positive indicator of homology for this table.

### 3.3. Deletion of VAC8 Affects Vacuolar Fusion

**Figure 2:**
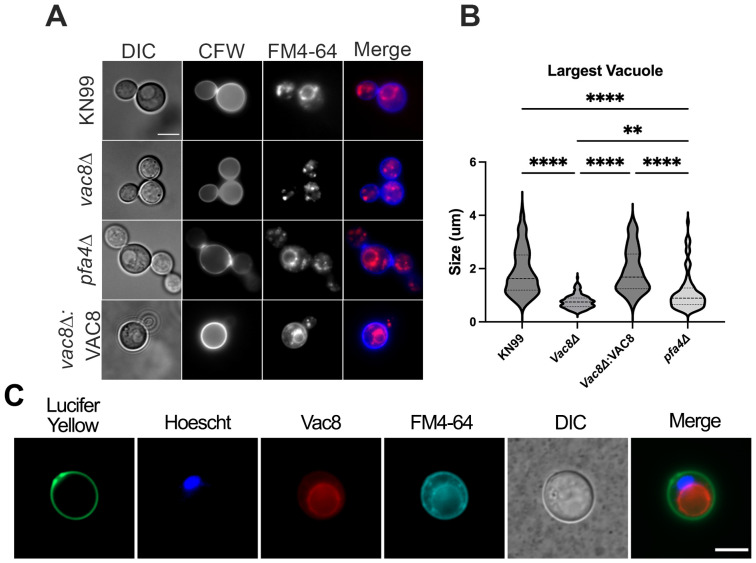
Vac8 regulates vacuole fusion. (**A**) Cells were cultured in YPD at 37 °C, supplemented with 1 M sorbitol, and stained with calcofluor white and FM4-64 to visualize the cell wall and vacuole, respectively. Scale bar: 5 μm. (**B**) Quantification of the largest vacuole in each cell by diameter (n: 84-246). *Vac8*Δ strains and *pfa4*Δ all show defects in ability to form a large vacuole under thermal and osmotic stress. (**C**) *vac8*Δ:*VAC8* cells grown in YPD and stained with Lucifer Yellow (green), Hoechst (blue), and FM4-64 (cyan) to visualize Vac8 (red) localization; vacuoles can also be seen with DIC imaging. Scale bar: 5 μm. Significance was assessed by 2-way ANOVA with multiple comparisons; ** *p* = 0.001 and **** *p* < 0.0001.

Since deletion of *PFA4* and *VAC8* results in fragmented vacuoles under nonstress conditions (YPD and 30 °C; Figure 1A), we hypothesize that *VAC8* might be playing a direct role in vacuolar fusion. To test this, we visualized vacuoles under conditions known to induce large vacuole formation in WT *C. neoformans* (YPD + 1 M sorbitol and 37 °C) [33]. We saw that WT *C. neoformans* KN99α has large, readily visible vacuoles in both FM4-64 and Differential Interference Contrast [42] imaging, while *pfa4*Δ has considerably smaller vacuoles, with relatively few cells generating a vacuole large enough to be seen in DIC (Figure 2A,B). Moreover, *vac8*Δ showed a marked inability to generate large vacuoles in response to high temperature and osmotic stress, with no obvious vacuoles being observed through DIC (Figure 2A,B). The average size of the vacuoles was compared between each strain by measuring the diameter of the largest vacuole as observed by FM4-64 staining and DIC. Loss of *VAC8* was sufficient to inhibit vacuole fusion under set conditions (Figure 2A,B). To further confirm the function of Vac8, we generated a complement (*vac8*Δ:*VAC8*) strain in the safe-haven 2 locus that contained an mRuby3 fluorescent tag at the C-terminus of *VAC8*. Not only were the *vac8*Δ:*VAC8* cells able to generate large vacuoles, but staining with Hoechst and FM4-64 showed that mRuby3 colocalizes with the FM4-64 stain of the vacuole, which remain distinct from the nuclear envelope (Figure 2C). This adds further support to the role of Vac8 in vacuolar functions, and further supports that the predicted lipid modifications are functional and attach Vac8 to the vacuolar membrane.

### 3.4. Loss of VAC8 Is Associated with Growth Defects Under Various Cell Stresses

**Figure 3:**
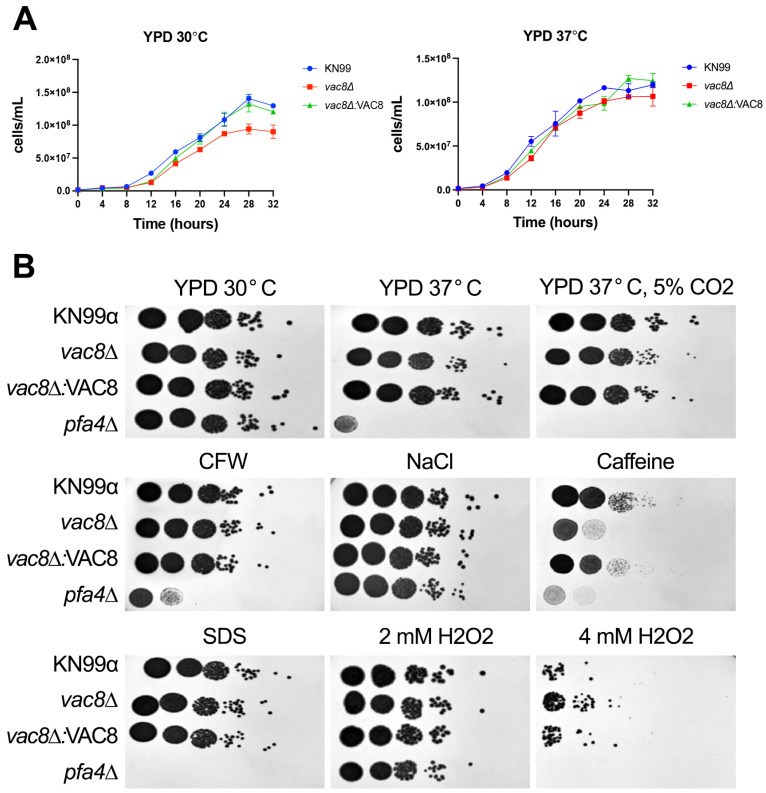
*vac8*Δ displays increased sensitivity to cell wall stressors and caffeine but increased resistance to oxidative stress. (**A**) Growth curves showing cell counts of KN99α, *vac8*Δ, and *vac8*Δ*:VAC8* grown in YPD at 30 or 37 °C for 32 h. (**B**) Serial dilutions (10^7^ to 10^3^) of cells were spotted on media containing the following stressors: 0.03% SDS, 2 or 4 mM H_2_O_2_, 0.5 mg/mL CFW, 1 M NaCl, 0.75 mg/mL caffeine, or YPD at various temperatures and conditions. All plates were incubated at 30 °C unless otherwise stated. Images shown are representative of at least three independent replicates.

Given the importance of normal vacuole function in a variety of cellular processes, *vac8*Δ was subjected to a series of cellular stressors (Figure 3B). All strains tested grew equally well on YPD agar at 30 °C. In *S. cerevisiae*, when the levels of Vac8 were reduced by swapping promoters, resulting in 10-fold-lower *VAC8* expression, there were differences in the cell wall composition, with increased levels of β-1,3-glucans and β-1,6-glucans, two of the major fungal cell wall components [40]. Therefore, we tested for cell wall defects by plating on calcofluor white (CFW), a cell wall stressor that binds to chitin. Interestingly, *vac8*Δ showed no defects on CFW. The *pfa4*Δ mutant, which lacks the palmitoyl acyl transferase for Vac8, displayed growth defects at high temperature (37 °C and 37 °C with 5% CO_2_), high salt (1 M NaCl), and caffeine (0.75 mg/mL caffeine) concentrations, as previously demonstrated [21]. The *vac8*Δ also showed significantly reduced growth at 0.75 mg/mL caffeine, suggesting an impaired response to general cell stress. Interestingly, growth on 2 mM H_2_O_2_ YPD plates was comparable to that of WT, but on 4 mM H_2_O_2_ plates, *vac8*Δ exhibited markedly increased growth, while *pfa4*Δ and KN99α growth was limited.

### 3.5. Main Virulence Factors Are Unaffected in vac8Δ Mutants

**Figure 4:**
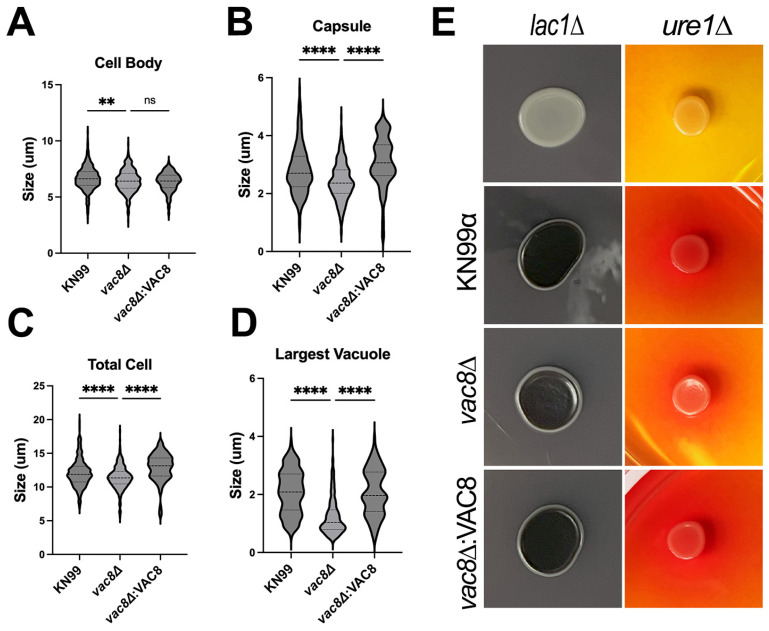
*vac8*Δ demonstrates a slight reduction in urease activity and reduced ability to form capsule which results in a reduced overall cell size. Quantification of cell body (**A**), capsule (**B**), total cell size (**C**), and largest vacuole (**D**) in capsule-inducing media (DMEM, 5% CO_2_, and 37 °C) after 48 h. Images were randomly taken and, for each measurement, at least 120 cells were analyzed. (**E**) Melanin production on L-DOPA plates (left) and urease production on Christensen’s urea agar plates [46] after 7 days. *lac1*Δ and *ure1*Δ serve as the negative controls for melanin and urease production, respectively. Significance was determined by one-way ANOVA with multiple comparisons. **, *p* < 0.005; ****, *p* < 0.0001.

Previous studies on *C. neoformans* and *C. albicans* have shown that normal vacuole function is needed for full virulence [11,12,43]. Similarly, in other human fungal pathogens like *C. glabrata* and *H. capsulatum*, normal vacuolar function has been implicated in the ability to cause disease through the vacuole’s role in autophagy and iron homeostasis [14,15]. To explore whether deletion of *VAC8* causes a defect in any of the main *C. neoformans* virulence factors [44], we tested growth at high temperature, capsule production, melanin production, and urease activity in WT and *vac8*Δ strains (Figure 3). The *vac8*Δ displays a slight decrease in growth on YPD agar at 37 °C and 37 °C with 5% CO_2_ suggesting it may have reduced fitness in an infection model. Additionally, *vac8*Δ shows slower growth rates at 30 °C relative to KN99α, although this is only noticeable in liquid media (Figure 3A). We further explored the effect of *vac8*Δ on the induction of the cryptococcal capsule, as this is a critical virulence factor involved in resisting the effects of the host innate immune system [45]. While some *C. neoformans* vacuolar mutants like *vph1*Δ do not produce capsule compared to WT [13], *vac8*Δ displays a modest but significant reduction in capsule, but still produced substantial levels of capsule (Figure 4A–C). Laccase activity was assessed by melanin production in media with L-DOPA, and all mutants displayed normal melanization (Figure 4E). Urease activity, determined by a phenol red-indicated pH change in the presence of urea, appeared reduced after 7 days in *vac8*Δ compared to KN99α and *vac8*Δ:*VAC8* (Figure 4E).

### 3.6. Vac8Δ Mutants Show Aberrant Budding and Impairment in Titanization

**Figure 5:**
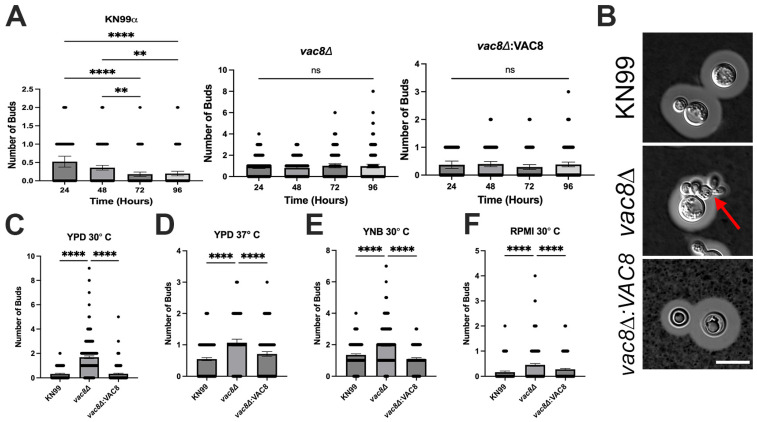
*vac8*Δ mutants show aberrant budding morphology in various growth conditions. (**A**) Quantification of the number of buds per cell in host-like conditions (DMEM, 37 °C, and 5% CO_2_) by fungal strain over time. (**B**) DIC images of wildtype and *vac8*Δ strains after 48 h in capsule-inducing conditions stained with India ink. Red arrow indicates elongated bud morphology. (**C**–**F**) Quantification of the number of buds per cell of fungal strains grown in various media conditions for 24 h. Data represent the mean and 95% confidence interval from at least 2 independent experiments and >175 cells quantified. Significance was determined by one-way ANOVA with multiple comparisons. ns, *p* > 0.05; **, *p* < 0.005; ****, *p* < 0.0001. Red arrow indicates abnormal budding morphology and elongated daughter cell shape. Scale bar: 10 μm.

Morphological transitions are a hallmark of fungal adaptations to host environments and play an important role in disease. In *C. neoformans*, formation of the polysaccharide capsule and titan cell formation are regulated by the cell cycle [47,48,49,50]. The cryptococcal capsule is one of the best characterized virulence factors of *C. neoformans*, and it is critical for infection, as acapsular mutants display severely attenuated virulence [45]. Under capsule-inducing conditions (37 °C + 5% CO_2_, DMEM, and 48 h), *vac8*Δ displayed increased levels of aberrant budding morphology with incomplete cytokinesis, including elongated cells and mother cells with multiple daughter cells branching from them (Figure 5). As incomplete cytokinesis is tied to abnormalities in the cell cycle, we sought to determine if the number of cells with abnormal budding morphologies increased over time. To explore this, we examined the bud morphology of cells grown in DMEM at 37 °C + 5% CO_2_ every 24 h for 96 h (Figure 5A). KN99α displays little to no cells with more than one bud at all timepoints examined. Interestingly, the percentage of cells with an aberrant budding morphology did not increase over time in *vac8*Δ, yet *vac8*Δ had significantly more budding defects compared to wildtype strains at each timepoint and media examined (Figure 5A,C–F).

**Figure 6:**
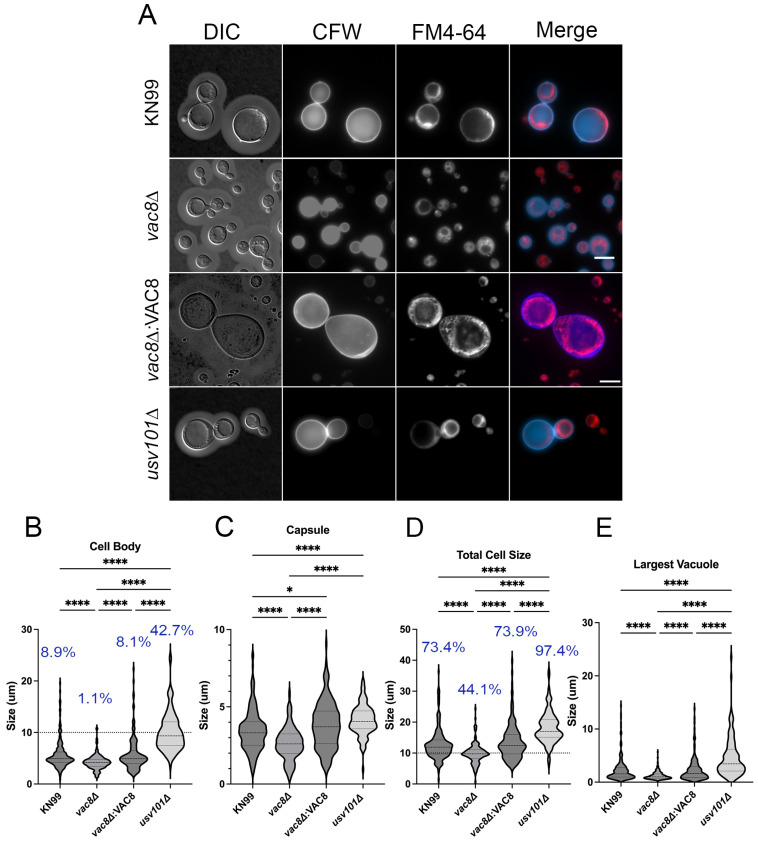
Loss of *VAC8* reduces the ability to form titan cells. (**A**) Images of cells grown in titan-inducing conditions (10% FBS, 5% CO_2_, and 37 °C) for 48 h. Cells were stained with calcofluor white (CFW) and FM4-64 to visualize the cell wall and vacuole structures, respectively. (**B**–**E**) Quantification of the cell body (**B**), capsule (**C**), total cell size (**D**) and the largest vacuole (**E**) in the mother cell. Scale bar is 10 μm. Dashed line represents 10 μm titan cell threshold. Blue percentages represent the percentage of cells > 10 μm. At least 115 cells were analyzed for each strain across 2 independent experiments. Significance was determined by ordinary one-way ANOVA with multiple comparisons. *, *p* < 0.05; ****, *p* < 0.0001.

Titan cells are a unique morphological state that can be induced in vivo by a mammalian lung environment and an internal *Galleria mellonella* environment [49,50,51]. These cells are characterized by their large size. Normal *C. neoformans* yeast cells have a cell body of 5–7 μm, while cell bodies of titan cells can reach up to 100 μm; an arbitrary cutoff of either 10 or 15 μm is commonly used for classification [52]. Additionally, these cells display increased ploidy, often tetraploid up to 128-ploid, compared to the haploid yeast; a large vacuole that encompasses the majority of intracellular space; a thickened cell wall (2–3 μm vs. 150–200 nm); and a denser and more cross-linked capsule [52]. These cells play a critical role during infection by contributing to fungal survival, the promotion of dissemination to the CNS, and involvement in latency and resistance to antifungal treatment [52,53]. Given that large vacuole formation is observed in titan cells and *vac8*Δ shows fragmented vacuoles, we sought to determine if *vac8*Δ can generate titan cells. Recently, multiple groups have identified ways to create bona fide titan cells in vitro using low-nutrient serum supplemented medium at a neutral pH with CO_2_ [49,50,51]. We utilized the method of Dambuza et al. [50] and a 10 μm threshold for the cell body as classification criteria for titan cells. Consistent with previous studies, ~10% of KN99α cells become titans, significantly less than *usv101*Δ at 42.7%, which readily forms titan cells (Figure 6A,B). Here, *vac8*Δ showed greatly reduced ability to form titan cells at 1.1%. Despite the inability to form titan cells, *vac8*Δ cells can produce a robust capsule, albeit smaller than KN99α and complement strains (Figure 6C). This led to WT strains and *usv101*Δ having significantly larger cells overall, approaching 40 μm, while very few *vac8*Δ cells reached a total cell size of 20 μm (cell body and capsule; Figure 6D).

Internally, titan cells produce a single large vacuole that may be involved in extracellular vesicle production [7]. It is unclear if the vacuoles of titan cells behave similarly or differ from that of typical cells. Here, we observed KN99α and *usv101*Δ typically display a single central vacuole that takes up the majority of the cell, which can easily be seen in DIC imaging or with the lipophilic dye FM4-64 (Figure 6A). In contrast, *vac8*Δ cells often show multiple smaller vacuoles that make up less of the internal environment, even in *vac8*Δ cells that would be considered titan cells. Given this, the ability to generate titan cells may be dependent on the ability to form a single large vacuole [10,12]. While the budding morphology was not quantified under these conditions, increased rates of incomplete cytokinesis were observed in the *vac8*Δ strain.

### 3.7. Loss of VAC8 Causes Decreased Susceptibility to Fluconazole

Due to the close evolutionary relationship between humans and yeast, there are few antifungal targets within yeast, which has resulted in the current limited number of antifungal drugs [2]. Most antifungal classes target the biosynthesis of ergosterol or the ergosterol molecule itself, the main sterol in fungal membranes [2,54]. While a newer class of antifungal, the echinocandins, target the biosynthesis of (1,3)-β-glucan, a key component of the fungal cell wall, it has limited applicability and is ineffective against *Cryptococcus* [2]. Therefore, there is a clear need to develop novel antifungal drugs specifically for *C. neoformans*. Alternatively, interventions that can augment the potency of current antifungal drugs, or restore sensitivity to them, would also be useful. Vacuoles play an important role in the detoxification and sequestration of compounds in yeast. Recently, they have been implicated in fluconazole resistance in *C. albicans* and *S. cerevisiae* through the ability of ABC transporters to import fluconazole into the vacuole [55,56,57]. Additionally, as FLC empties the vacuolar membranes of ergosterol, or AmB binds to ergosterol in that location, it inhibits V-ATPase complex formation, preventing the vacuole from acidifying [58,59]. Thus, targeting the vacuole might be a way to modulate the potency of current antifungals, thereby increasing their efficacy.

**Figure 7:**
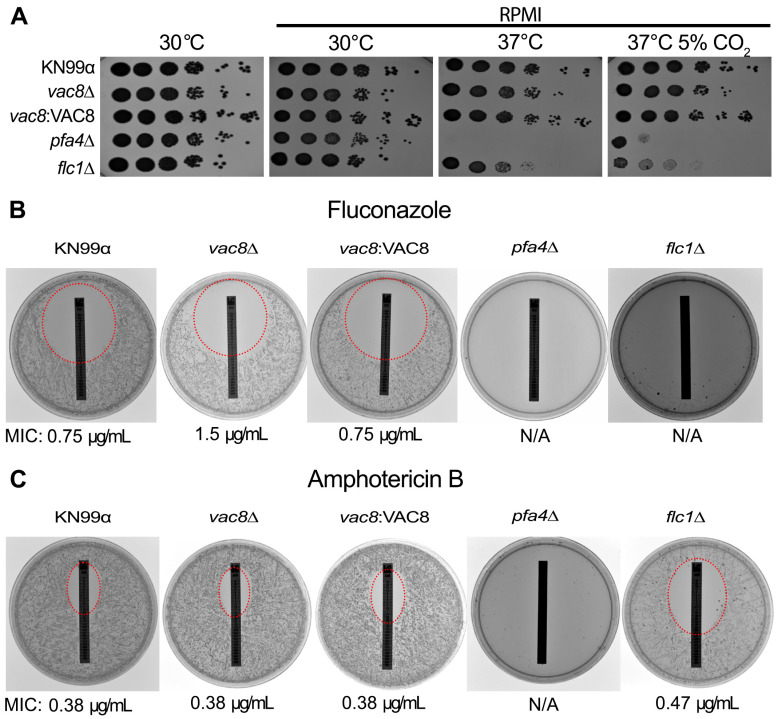
*vac8*Δ strains show a slight increase in resistance to fluconazole but no changes to polyene susceptibility despite a slight sensitivity to high temperature and CO_2_ growth. (**A**) The indicated strains were plated in serial dilution and grown on YPD at 30 °C, or RPMI plates and incubated at 30 °C, 37 °C, or 37 °C with 5% CO_2_ for 72 h before being imaged. (**B**,**C**) Fluconazole (FLC; **B**) or amphotericin B (AmB; **C**) Epsilometer test strips were applied to RPMI 1640 agar plates with 2  ×  10^5^ cells of the indicated strain. The zone of inhibition is highlighted with a red dashed circle where applicable, with the MIC listed below the image (as determined by where the dashed line crosses the E-test strip). The MICs of KN99α and *vac8*Δ mutants were all 0.19 μg/mL. *pfa4*Δ shows complete inhibition of growth in the presence of both FLC and AmB, while *flc1*Δ is also completely inhibited by FLC; however, AmB shows an MIC of 0.47 μg/mL. These plates were incubated for 72  h at 37 °C and 5% CO_2_. Images shown are representative of 3 independent experiments.

To this end, we examined if *vac8*Δ and other mutants with abnormal vacuolar morphologies (*pfa4*Δ and *flc1*Δ) display increased susceptibility to the two most common antifungals: FLC (azole class) and AmB (polyene). First, cells were incubated under optimal conditions (YPD and 30 °C) to determine if an aberrant vacuole morphology affects growth under these conditions (Figure 7A). While no strain showed impaired growth on YPD or at 30 °C, growth at high temperature on RPMI plates was impaired in *vac8*Δ, *pfa4*Δ, and *flc1*Δ. Interestingly, the addition of CO_2_ slightly recovered the growth of *pfa4*Δ while not markedly changing the relative growth of *vac8*Δ or *pfa4*Δ (Figure 7A). We examined the susceptibility to antifungal cells grown on RPMI agar plates with an E-test strip for fluconazole or amphotericin B. After 72 h, minimum inhibitory concentrations (MICs) were determined by examining where the edge of the zone of inhibition crosses the test strip. Interestingly, the *vac8*Δ strain showed a modest decrease in susceptibility to fluconazole, with MICs of 1.5 μg/mL compared to 0.75 μg/mL for KN99α (Figure 7B). The MIC for AmB remained consistent at 0.38 μg/mL (Figure 7C).

### 3.8. Infection with vac8Δ Does Not Alter Disease Outcome in a Murine Model

**Figure 8:**
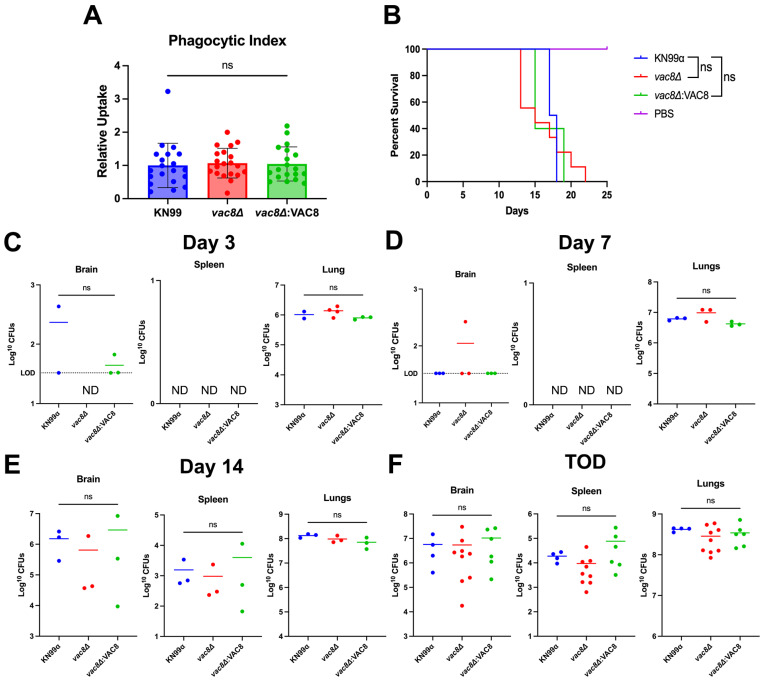
*C neoformans vac8*Δ-infected mice succumb to infection at similar rates to KN99α-infected mice. (**A**) Phagocytic index of THP-1 macrophages infected with the indicated fungal strains at an MOI of 1. Significance was determined using an ordinary one-way ANOVA with multiple comparisons. (**B**) In vivo survival of 5-to-6-week-old female A/J mice infected with KN99α, *vac8*Δ, *vac8*Δ:*VAC8,* or DPBS (mock) and monitored for 25 days when the experiment was terminated. A total of 4–9 animals were used per group. Significance was determined using Mantel–Cox test. (**C**–**F**) Quantified organ burdens of KN99α-, *vac8*Δ-, and *vac8*Δ:*VAC8*-infected mice at 3, 7, and 14 days post-infection (2–4 animals per group per timepoint), and at time of death. Organs were harvested and homogenized, and dilutions were plated on YPD plates. Plates were incubated for 48 h and CFUs were counted. Each data point represents a mouse, and black bars represent the mean. Significance was determined using an ordinary one-way ANOVA with multiple comparisons. ns, *p* > 0.05.

Innate lung immune cells, in particular alveolar macrophages, represent the first line of defense against cryptococcal infections, and the complex fungal–macrophage interaction will ultimately help determine the outcome of the infection [60]. The *vac8*Δ mutant has previously been reported as a high-uptake mutant in both murine bone marrow and human blood-derived macrophages [61]. To determine if *vac8*Δ cells display different interactions with macrophages in vitro, cells were incubated with THP-1 macrophages (a model for alveolar macrophages) for one hour, and the phagocytic index was determined, as previously reported [21]. There was no difference in the uptake of KN99α, *vac8*Δ, and *vac8*Δ:*VAC8* cells by THP-1 cells (Figure 8A). To further explore to role of *vac8*Δ in *C. neoformans* pathology, 5-to-6-week-old A/J mice were infected with 5 × 10^5^ fungal cells via intranasal inoculation. Mice infected with *vac8*Δ started to succumb to infection prior to KN99α- and *vac8*Δ:*VAC8*-infected mice, but mean survival was not statistically different (Figure 8B). Given the difference in responses to cellular stressors, fungal organ burden was determined at 3, 7, and 14 days post-infection (dpi). At 3 and 7 dpi, no strain had disseminated to the spleen (Figure 8C,D). At 3 dpi, both KN99α- and *vac8*Δ:*VAC8*-infected mice showed low levels of dissemination to the brain, while only the *vac8*Δ-infected mice showed low levels of dissemination at 7 dpi (Figure 8C,D). Fungal organ burden in the brain, spleen, and lung was similar amongst mice infected with each strain at all timepoints (Figure 8C–F).

### 3.9. Infection with vac8Δ Alters the Lung Environment Despite Not Impacting Disease Progression

**Figure 9:**
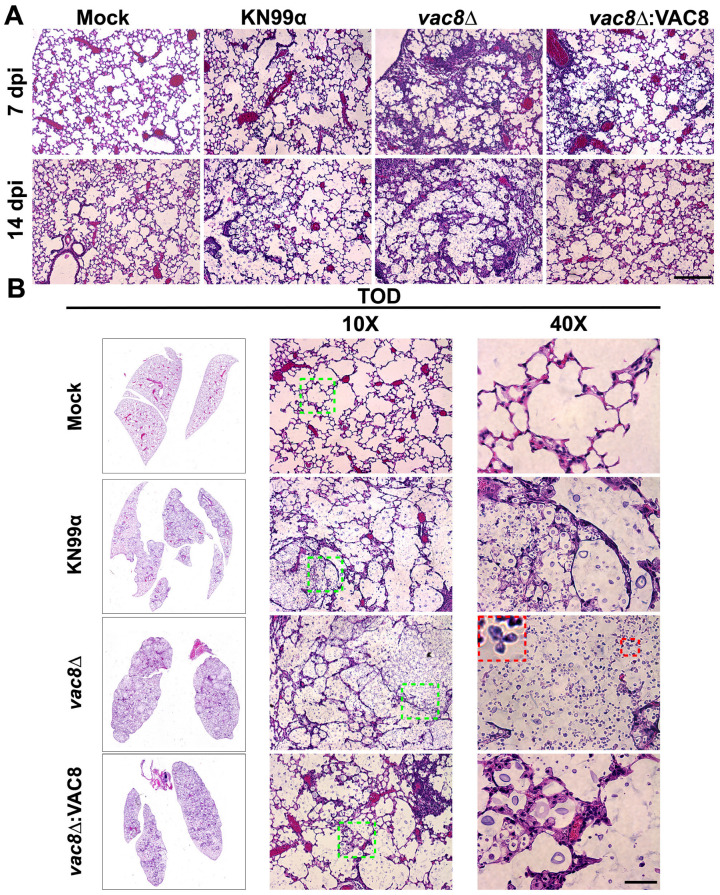
Infection with *C. neoformans vac8*Δ results in small differences in histopathology. (**A**) Dissected lungs of female A/J mice infected with 5 × 10^4^ cells of the KN99α wildtype strain, *vac8*Δ, or *vac8*Δ:*VAC8* strain, or DPBS-infected (mock), were harvested at predetermined endpoints of 7 and 14 dpi. (**B**) Gross organ examination revealed significant changes in inflammation and tissue damage at time of death. H&E staining was used to visualize microscopic lung pathology. Green boxes on 10× images are inset as 40×. Red boxes highlight abnormal fungal morphology observed in the lungs of mice infected with the *vac8*Δ strain. The 40× scale bar is 50 μm [64].

*Cryptococcus* initially colonizes the lung environment, yet the fatal pathology is associated with dissemination to the central nervous system and meningoencephalitis [62,63]. Therefore, the lung environment is critical in controlling the overall course of infection. To qualitatively examine any differences at the tissue and cellular levels, lungs of mock-, KN99α-, *vac8*Δ-, and *vac8*Δ:*VAC8*-infected mice were harvested and stained with hematoxylin and eosin (H&E). By 7 dpi, the lungs of *vac8*Δ-infected mice showed increased pockets of inflammation that were absent from the lungs of mice infected with KN99α, *vac8*Δ:*VAC8*, or mock (Figure 9A). This trend continued at 14 dpi, with all infected lungs displaying some level of tissue damage (Figure 9A). Despite not seeing differences in organ burden by CFUs, *vac8*Δ-infected lungs showed pockets of inflammation near blood vessels and large nodules in the lung, where all healthy tissue had been destroyed, leaving large pockets filled with yeast (Figure 9B). This is in contrast to the lungs of KN99α- and *vac8*Δ:*VAC8*-infected mice that, despite similar levels of fungal burden, show minimal levels of inflammation, a characteristic of cryptococcosis (Figure 9B). Interestingly, the yeast observed in KN99α- and *vac8*Δ:*VAC8*-infected lungs are mostly single-celled, and very few appear to have a bud, while *vac8*Δ cells in the lungs display the same aberrant morphologies seen in vitro (Figure 5 and Figure 9B, red inset).

## 4. Discussion

Here, we report an initial characterization of vacuolar protein 8 of *C. neoformans,* and its role in vacuolar and cellular morphologies in response to cell stress. First, we confirmed that CnVac8 is a true homolog of ScVac8, which is predicted to have conserved myristoylation and palmitoylation sites at the N-terminus to allow for association onto the vacuolar membrane, and vacuolar defects when deleted (Figure 1). Additionally, Interpro and AlphaFold predictions reveal a protein structure very similar to the ScVac8 crystal structure, with a central superhelix comprised of 11 ARM repeats, serving as a protein-binding platform, an intrinsically disordered region near the N-terminus with glycine and cysteine residues, and a large, disordered loop at the C-terminus. Despite a large evolutionary distance between ascomycetes, basidiomycetes, and Mucorales species, Vac8 has a well-conserved protein structure that explains the overlapping and distinct roles it plays. Further studies should be carried out to confirm palmitoylation and myristoylation sites and determine their role in lipid modifications to CnVac8 functions.

In *S. cerevisiae*, Vac8 is well characterized and has multiple binding partners that facilitate its involvement in key cellular processes, like piecemeal autophagy of the nucleus via the nuclear–vacuole junction, autophagy via Atg13, and vacuolar inheritance via Vac17. Given the evolutionary distance between *S. cerevisiae* and *C. neoformans*, we sought to determine if CnVac8 would bind with homologs of ScVac8 binding partners to carry out the same cellular functions in *C. neoformans*. Unexpectedly, BLAST-P and BLAST-PSI searches of the *C. neoformans* proteome for ScVac8 binding partners revealed few homologs for any of the known proteins (Table 1). This suggests that, despite the conserved protein structure of ScVac8 and CnVac8, their functions may diverge through interactions with unique binding partners. Absence of a Vac17 homolog, the adaptor linking ScVac8 to ScMyo2 for vacuole movement and inheritance, may explain the apparently normal vacuole inheritance in *C. neoformans vac8*Δ. This is not surprising if we consider that, in other basidiomycetes such as *Ustilago maydis*, vacuolar movement and inheritance is kinesin-dependent rather than myosin-dependent, like in *S. cerevisiae* [65]. Thus, identifying the CnVac8 binding partners might reveal a novel biology applicable to other basidiomycetes.

The fungal vacuole is a unique organelle with a diverse set of functions, including protein degradation, ionic and metabolite storage, osmoregulation, and regulation of intracellular pH [64]. Given that many of these functions are needed for full virulence in other pathogens, and the need to find novel, fungal-specific drug targets, we next assessed if the *vac8*Δ strains exhibit defects under common cell stressors (Figure 3). The absence of a closely related organelle to the vacuole in mammals indicates that it may be an advantageous target for the development of future therapeutics. Additionally, *vac8*Δ grew similarly to the WT at room temperature, under osmotic stress, nitrosative stress, and with the cell wall stressor CFW. They also displayed modest growth defects at high temperature (37 °C), high temperature with CO_2_, and cell membrane stress (SDS). Conversely, *vac8*Δ showed a significant growth impairment on media supplemented with caffeine, suggesting *vac8*Δ may be involved in a general cell stress pathway. Surprisingly, at 2 mM hydrogen peroxide, *vac8*Δ exhibits normal growth, but when exposed to higher levels of 4 mM hydrogen peroxide, it displays increased growth compared to WT and *pfa4*Δ. Studies of superoxide dismutase (SOD) mutants in *C. neoformans*, which lead to higher intracellular reactive oxygen species or via menadione treatment, showed increased vacuolar fragmentation [66]. Therefore, we hypothesize alongside others that vacuolar fragmentation increases the surface-to-volume ratio, and allows for more iron transporters to bind and increase iron uptake, which serves to protect cells from oxidative stress [66,67]. This same hypothesis might apply to the interesting observation that *vac8*Δ mutants are slightly more resistant to FLC without any change in susceptibility to AmB (Figure 7). Recent evidence in *C. albicans* suggests that vacuolar sequestration of azoles through the ATP-binding cassette (ABC) transporter Mlt1p contributes to azole resistance [68]. Consistently, deletion of Mlt1p in *C. albicans* (or its homolog in *S. cerevisiae* Ybt1p) leads to azole susceptibility [68]. Similar to the yeast vacuole response to oxidative stress, vacuole fragmentation may play a beneficial role in responding to challenge from azoles and potentially facilitate increased sequestration. Supporting this idea is the recent finding that, in *C. neoformans*, treatment of cells with fluconazole leads to increased vacuolar fragmentation [66].

Since previous vacuolar mutants, like *vph1*Δ, which encodes a subunit of the vacuole H^+^-ATPase, are defective in capsule production, laccase production, and growth at 37 °C, we also assessed these virulence factors in *vac8*Δ [13]. Despite an aberrant vacuole morphology, *vac8*Δ shows a modest reduction in capsule production (Figure 4). Both laccase and urease production were similar to WT strains, demonstrating that some, but not all, virulence factors were affected by the deletion of *VAC8*. These phenotypes could be explained by the fact that, although the vacuole is fragmented in *vac8*Δ, it may still be functional, while in the *vph1*Δ, the vacuole is not functional [13].

While examining the ability of *vac8*Δ to produce capsule we noted an increase in aberrant cell morphologies, including multi-budded cells and cells with small, elongated buds (Figure 5). We sought to determine if the number of partially formed buds increased over time, as that may indicate issues with cell cycle regulation, possibly due to defective vacuole migration into daughter cells [69]. In *S. cerevisiae*, the absence of vacuole migration from mother to daughter cells is not lethal under normal conditions because the daughter cell can generate a new vacuole “de novo” through unknown mechanisms [34]. However, it still appears a functional vacuole is required for cell cycle progression out of G1 phase; thus, any defects in vacuole migration or function may result in cell cycle arrest [69]. This migration is an actin-dependent process using the molecular motor Myo2 in complex with Vac17 and Vac8 [34]. Given that we did not find a homolog of Vac17, we were uncertain if CnVac8 would be involved in the migration of the vacuole and produce delay in growth. The number of cells displaying abnormal budding did not increase significantly over time in wildtype or *vac8*Δ strains. However, *vac8*Δ showed much higher levels of incomplete cytokinesis and abnormal bud morphologies, with very few WT cells having more than one daughter cell (Figure 5A). Despite not increasing over time, it is clear *VAC8* and normal vacuolar function are required for normal budding behavior.

**Figure 10:**
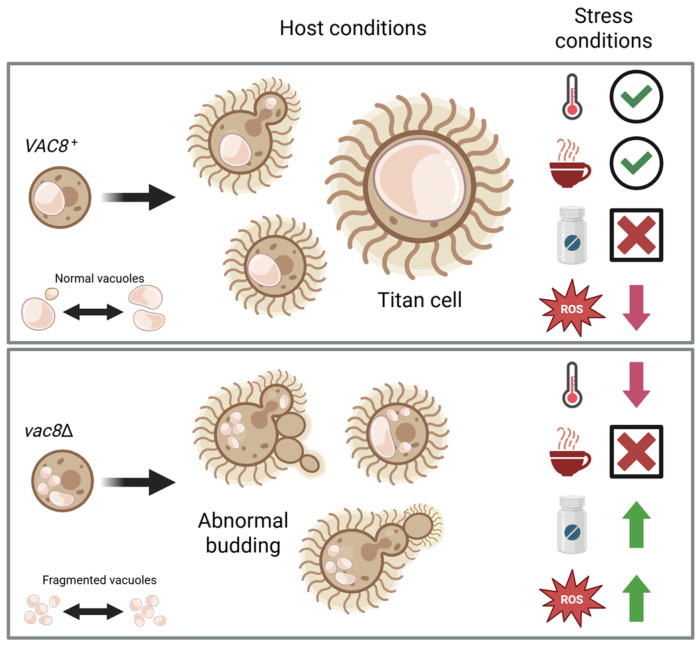
A model depicting the role of Vac8 in *C. neoformans* biology. Vac8 contributes to various cellular functions by promoting homotypic vacuole fusion or enlargement. In conditions that initiate vacuolar fusion or enlargement, such as during titan cell formation, cells lacking Vac8 display only small fragmented vacuoles and are unable to titanize, while cells with Vac8 are able to generate 1 or 2 large vacuoles and readily form titan cells. Additionally, the absence of Vac8 leads to aberrant budding morphology or incomplete cytokinesis under various growth conditions, which may explain the mild growth defect at higher temperatures (red thermometer symbol). We believe that this fragmentation may also explain the sensitivity to caffeine (coffee cup symbol), and the increased resistance to fluconazole (pill bottle symbol) and H_2_O_2_ (ROS-labeled red star). Green checkmark and red cross represent normal and no growth, respectively; the green and red arrows represent increased or decreased growth, respectively. Image created with BioRender (https://biorender.com/uxd3087).

Along this same line, vacuole size helps control cell size in yeast, as well as the initiation of bud formation, and this may also play a role in titan cell formation [52,69]. Using previously established in vitro methods, we induced titan cell formation and quantified the cell body, capsule, and total cell size (Figure 6). While *vac8*Δ and WT strains were able to produce robust capsule, *vac8*Δ showed severely limited ability to form cells >10 μm. Examining the vacuole in these titan cells using FM4-64 and DIC highlighted the very large vacuoles in the WT cells, with a single vacuole encompassing nearly all of the internal space. In contrast, *vac8*Δ cells often have multiple vacuoles that do not encompass large portions of the cell body. In some cases, there is one larger vacuole with one or more smaller vacuoles. Together, this suggests *VAC8* plays a role in facilitating vacuole–vacuole fusion and/or vacuole enlargement, and the impairment of this process limits cell body enlargement into titan cells (Figure 10). Given that titanization is also related to the cell cycle [70], it is not surprising that, under these conditions, we also see the abnormal budding morphology in *vac8*Δ mutants, supporting the idea that, in *C. neoformans,* there is also a vacuolar cell cycle checkpoint.

Lastly, given the cellular and growth defects under certain conditions related to virulence, such as inability to titanize, it was surprising that we found that Vac8 function is not necessary for normal disease progression in a murine model of cryptococcosis (Figure 8 and Figure 9). This may be partially explained by *vac8*Δ’s increased resistance to oxidative stress, which the yeast is likely to encounter in a host. Also, we hypothesize the absence of *VAC8* leads to increased vacuole fragmentation but does not impair overall vacuolar function. This may explain the increased resistance to oxidative stress and fluconazole and slight growth defects while remaining capable of causing disease. This shows CnVac8 plays a similar role to ScVac8 in regard to membrane tethering and homotypic vacuolar fusion, but also new roles in cell morphology, leading to abnormalities in cell budding and defective titanization. Because of these unique functions, *VAC8* might still be needed for normal progression and disease manifestation in a mammalian host, similar to how in *C. albicans* vacuole enlargement is needed to promote invasive hyphal growth [71]. Notably, to our knowledge, this is the first mutant with defective titanization that is not attenuated for virulence.

Uncovering a role for *VAC8* in *C. neoformans* biology has highlighted an important role of the fungal vacuole in cellular responses to various stresses, including exposure to antifungals and host environments (Figure 10). Sc*VAC8*, in particular, has been extensively studied in model yeast because, in *S. cerevisiae*, it is the only ARM repeat-containing protein. ARM repeats are conserved in eukaryotes, but with distinct and overlapping cellular roles. This diverse list of roles is because ARM repeat-containing proteins can bind multiple partners, contributing to multiple functions. Future research on Cn*VAC8* will unveil some of these additional functions; however, through a better understanding of the role of *VAC8* in cryptococcal vacuoles, we ultimately hope to identify the processes and potential targets that may make these fungal pathogens more susceptible to antifungal therapies, while also uncovering novel cell biology.

## Data Availability

All data are presented within this article or Supplementary Materials. For the DNA sequence of the CRISPR constructs and/or the raw sequencing results, please contact the authors directly.

## Supplementary Materials

The following supporting information can be downloaded at: https://www.mdpi.com/article/10.3390/jof11120877/s1, Figure S1: Loss of *VAC8* induces aberrant budding in host-like conditions; and Table S1: Vac8 protein modification predictions.
