# Supplementary material for: The Vacuolar Protein 8 (Vac8) Homolog in Cryptococcus neoformans Impacts Stress Responses and Virulence Traits Through Conserved and Unique Roles
Source: J Fungi (Basel). 2025 Dec 11;11(12):877. doi: 10.3390/jof11120877 (PMC12734321; doi:10.3390/jof11120877)
Supplement: Supplementary file 1 [file jof-11-00877-s001.zip › Supplemental Figure S1.pdf]

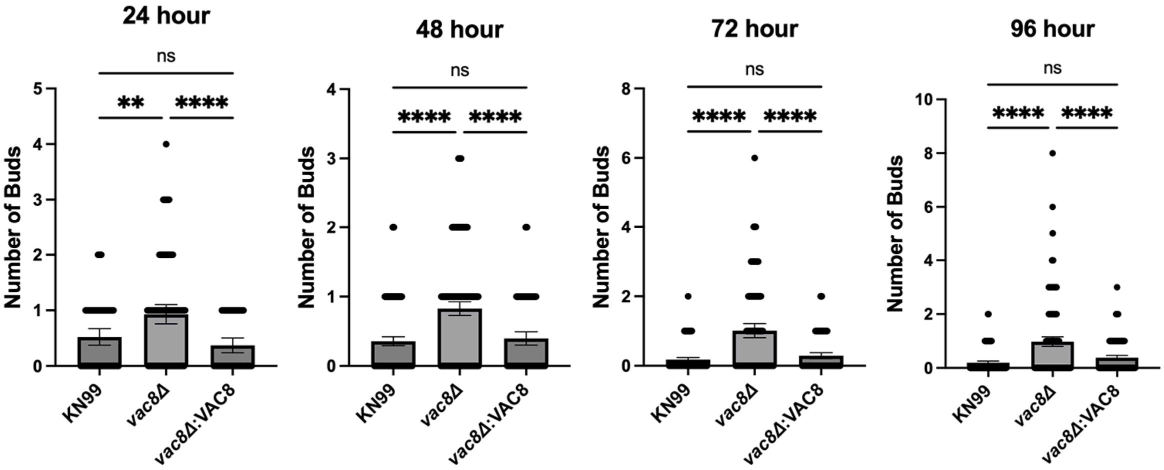

Supplemental Figure S1: Loss of *VAC8* induces aberrant budding in host like conditions. Quantification of the number of buds per cell grown in DMEM, 37°C and 5% CO_2 for 24, 48, 72 and 96 hours. Data represents the mean and 95% confidence interval from 2 independent experiments and >175 cells. Significance was determined by one-way ANOVA with multiple comparisons. ****, p<0.0001.
