# Supplementary material for: The Vacuolar Protein 8 (Vac8) Homolog in Cryptococcus neoformans Impacts Stress Responses and Virulence Traits Through Conserved and Unique Roles
Source: J Fungi (Basel). 2025 Dec 11;11(12):877. doi: 10.3390/jof11120877 (PMC12734321; doi:10.3390/jof11120877)
Supplement: Supplementary file 1 [file jof-11-00877-s001.zip › Supplemental Table S1.pdf]

Species (Strain name)	FungiDB gene ID	Protein Length (aa)	Palmitoylation prediction aa (Score)	Myristylation Prediction aa (Score)
Cryptococcus gattii (R265)	CNBG_0209_t1	628	8 (0.9714)<br>9 (0.9667)	2 (.9951)
Cryptococcus gattii (WM276)	CGB_B4090W-t26_1	628	8 (0.9714)<br>9 (0.9667)	2 (.9951)
Cryptococcus deneoformans (JEC21)	CNA03500-t26_1	630	8 (.9701)<br>9 (.9643)	2 (.9951)
Cryptococcus neoformans (H99)	CNAG_00354-t26_1	628	8 (.9701)<br>9 (.9643)	2 (.9951)
Kwoniella bestiolae (CBS 10118)	I302_05199	624		N/A
Coprinopsis cinerea okayama	CC1G_13680-t26_1	619	7 (.9459)	2 (.9928)
Ustilago maydis (521)	UMAG_15050_t1	560	5 (.9677)<br>7 (.9396)<br>8 (.9300)<br>540 (.9471)	2 (.9572)
Mucor circinelloides (1006PhL)	HMPREF1544_05625-t46_1	570	5 (.9537)<br>8 (.9466)<br>9 (.9139)	2 (.9930)
Rhizopus delemar (Formerly oryzae) (RA 99-880)	RO3G_12596-t26_1	566	N/A	N/A
Saccharomyces cerevisiae (S288C)	YEL013W-t26_1	578	4 (.9695)<br>5 (.9679)<br>7 (.9012)<br>445 (.9062)	2 (.9928)
Candida auris (B8441)	B9J08_004608-t37_1	558	4 (.9527)<br>5 (.9451)	2 (.6369)
Candida albicans (SC5314)	C4_05150W_A-T	585	4 (.9684)<br>5 (.9607)<br>7 (.9290)	2 (.9709)
Pneumocystis jirovecii (SE8)	PNEJI1_002355-t26_1	565		2 (.0076)
Hortaea wernickii (EXF-2000)	BTJ68_12171_t1	586	5 (.9305)<br>12 (.9472)<br>13 (.9332)<br>274 (.9040)	N/A
Fusarium solani (FSSC 5 MPI-SDFR-AT-009)	B0J15DRAFT_583410_t1	559	4 (.9732)<br>8 (.9210)<br>9 (.8992)	2 (.0392)
Neuorspora crassa (OR74A)	NCU01470-t26_1	559	5 (.9724)<br>8 (.9504)<br>9 (.9332)	2 (.3589)
Sporothrix brasiliensis (5110)	SPBR_05719-t41_1	579	4 (.9757)<br>8 (.9524)<br>9 (.9255)	2 (.0392)
Aspergillus fumigatus (Af293)	Afu5g13540-T	578	8 (.9447)<br>563 (.9250)	N/A

Cladophialophora bantiana (CBS 173.52)	Z519_08659_t1	562	8 (.9411)<br>9 (.9135)	2 (.9950)
Exophiala oligosperma CBS 72588	PV06_08765-t43_2	562	9 (.9583)	2 (.9951)
Blastomyces dermatitidis (ER-3)	BDCG_05075_t1	560	5 (.9703)<br>8 (.9633)	2 (.0147)
Paracoccidiodes brasiliensis (Pb03)	PABG_05843-t30_1	587	5 (.9491)<br>8 (.9574)	2 (.1323)
Microsporum canis (CBS 113480)	MCYG_03982_t1	557	5 (.9708)<br>8 (.9646)	2 (.0211)
Coccidioides posadasii (strain Silveira)	CPSG_03901-t26_1	558	5 (.9708)<br>8 (.9667)	2 (.0153)
Coccidioides immitis (RS)	CIMG_09351-t26_1	578	5 (.9410)<br>8 (.9387)	2 (.1773)
Histoplasma capsulatum (G217B)	I7I48_00544_t1	580	5 (.9534)<br>8 (.9544)	2 (.2121)

Supplemental Table S1*:* Vac8 protein modification predictions. Protein length and predicted lipid modification with amino acid residues. Confidence threshold of 0.9000 was used as a positive indicator for both palmitoylation and myristoylation.[26, 27] N/A: there is no N-terminal glycine residue for myristylation.
